# Interactions of highly and low virulent *Flavobacterium columnare* isolates with gill tissue in carp and rainbow trout

**DOI:** 10.1186/s13567-015-0164-5

**Published:** 2015-03-06

**Authors:** Annelies Maria Declercq, Koen Chiers, Wim Van den Broeck, Jeroen Dewulf, Venessa Eeckhaut, Maria Cornelissen, Peter Bossier, Freddy Haesebrouck, Annemie Decostere

**Affiliations:** Department Morphology, Ghent University, Faculty of Veterinary Medicine, Salisburylaan 133, 9820 Merelbeke, Belgium; Department of Pathology, Bacteriology and Poultry diseases, Ghent University, Faculty of Veterinary Medicine, Salisburylaan 133, 9820 Merelbeke, Belgium; Department of Reproduction, Obstetrics and Herd Health, Ghent University, Faculty of Veterinary Medicine, Salisburylaan 133, 9820 Merelbeke, Belgium; Department of Basic Medical Sciences, Faculty of Medicine and Health Sciences, Ghent University, De Pintelaan 185, 9000 Ghent, Belgium; Ghent University, Laboratory of Aquaculture and Artemia Reference Center, Rozier 44, 9000 Ghent, Belgium

## Abstract

The interactions of *Flavobacterium columnare* isolates of different virulence with the gills of carp (*Cyprinus carpio* L.) and rainbow trout (*Oncorhynchus mykiss* Walbaum) were investigated. Both fish species were exposed to different high (HV) or low virulence (LV) isolates and sacrificed at seven predetermined times post-challenge. Histopathological and ultrastructural examination of carp and rainbow trout inoculated with the HV-isolate disclosed bacterial invasion and concomitant destruction of the gill tissue, gradually spreading from the filament tips towards the base, with outer membrane vesicles surrounding most bacterial cells. In carp, 5-10% of the fish inoculated with the LV-isolate became moribund and their gill tissue displayed the same features as described for the HV-isolate, albeit to a lesser degree. The bacterial numbers retrieved from the gill tissue were significantly higher for HV- compared to LV-isolate challenged carp and rainbow trout. TUNEL-stained and caspase-3-immunostained gill sections demonstrated significantly higher apoptotic cell counts in carp and rainbow trout challenged with the HV-isolate compared to control animals. Periodic acid-*Schiff*/alcian blue staining demonstrated a significantly higher total gill goblet cell count for HV- and LV-isolate challenged compared to control carp. Moreover, bacterial clusters were embedded in a neutral matrix while being encased by acid mucins, resembling biofilm formation. Eosinophilic granular cell counts were significantly higher in the HV-isolate compared to LV-isolate inoculated and control carp. The present data indicate a high colonization capacity, and the destructive and apoptotic-promoting features of the HV-isolate, and point towards important dynamic host mucin–*F. columnare* interactions warranting further research.

**Electronic supplementary material:**

The online version of this article (doi:10.1186/s13567-015-0164-5) contains supplementary material, which is available to authorized users.

## Introduction

Columnaris disease, caused by the Gram-negative bacterium *Flavobacterium columnare*, is notorious in freshwater aquaculture, amongst others of carp (*Cyprinus carpio* L.) and rainbow trout (*Oncorhynchus mykiss* Walbaum), in which it induces severe economic losses due to gill, skin and fin lesions often resulting in high mortality [1-10]. Recently, the bacterium-host interactions of columnaris disease were reviewed, whereby the various prevailing knowledge gaps were highlighted [11]. The mechanisms adopted by the pathogen to establish itself and to maintain a grip on the skin and the gill tissue, and consequently to elicit disease and mortality, are far from fully elucidated. Especially the interplay of *F. columnare* with the gill tissue still puzzles the research community. Hitherto, only a few studies explored the interaction between *F. columnare* and the gill tissue [12-15] focussing on host mucosal responses. Sun et al. studied the transcriptomic profiling of host responses in the gill tissue to columnaris disease following experimental challenge in catfish and found a rhamnose-binding lectin with putative roles in bacterial attachment and aggregation, and several immune suppressive pathways being stimulated after infection with *F. columnare* [15]. Accordingly, Peatman et al. found resistant catfish to have a higher expression of immune stimulating genes in the gills following challenge with *F. columnare* as compared to susceptible fish which showed high expression levels of a rhamnose-binding lectin and several mucosal immune suppression factors, possibly predisposing them to *F. columnare* infection [14].

In a recent study, variation in virulence between different *F. columnare* strains isolated from carp and rainbow trout was shown and the highly virulent isolates induced severe gill lesions in experimentally infected carp and rainbow trout [16]. The carp showed a diffuse lesion pattern, affecting all gill arches bilaterally and the animals died within 12 h after inoculation. In rainbow trout, the distribution pattern of the gill lesions was more focal and only present in the first gill arches. Mortality started 15 to 18 h after inoculation, also reaching 100% within 72 h.

To obtain better insights in the interaction of *F. columnare* isolates of differential virulence with the gills of carp and rainbow trout, the sequence of events taking place at the level of the gill tissue following challenge with a highly and a low virulent isolate was mapped. Gill health status, pathogen localisation and spread, degree of apoptosis, changes in chloride cell number, quantitative and qualitative mucus changes and bacterial cell counts were investigated at seven predetermined sampling points post-challenge. By merging the retrieved data, we sought to further elucidate the *F. columnare*-gill interplay.

## Materials and methods

### Fish

Two-day old carp fry were obtained from a Belgian hatchery and grown to a mean length of six centimetres before inclusion in the experiment. Rainbow trout with an average length of five centimetres were purchased from a Belgian hatchery (Villers-le-Gambon, Belgium) and acclimatized for one month in our facilities. The fish were maintained in one cubic metre stocking tanks filled with 800 L of recirculating and aerated tapwater. The water temperature was 22 ± 1 °C for the carp and 19 ± 1 °C for the rainbow trout. Starting from two weeks before the experimental challenge, the water temperature of the stocking tanks was gradually increased by 1 °C every two days until a temperature of 25 ± 1 °C and 22 ± 1 °C was reached for the carp and rainbow trout, respectively. This water temperature was then kept constant until the onset of the challenge. Free and ionized ammonia and nitrite concentrations were determined daily and were below detectable levels at all times. A photoperiod of 12 h light/12 h darkness was provided and the fish were fed a commercial diet (rainbow trout: Trouw Nutrition, carp: Fin Perfect Feed, Sonubaits) to satiation twice daily. Fish were deprived from food 24 h prior to the experimental challenge. Twenty-five carp and twenty-five rainbow trout were sacrificed with an overdose of benzocaine (ethylaminobenzoate; Sigma, Belgium; 10 g/100 mL acetone). Parasites were not observed during microscopic examination of wet mount preparations, made from scrapings of the gill and skin tissue. Gill and skin were also screened for the presence of *F. columnare* by means of Polymerase Chain Reaction (PCR) and bacteriological examination using cultivation onto modified Shieh agar [17,18] containing 1 μg/mL tobramycin [19]. For the PCR, DNA from the tissue samples was extracted using a DNeasy blood and tissue kit (Qiagen, Venlo, the Netherlands), according to the guidelines of the manufacturer. PCR mixtures, primer sequences and cycle conditions were as described before [20,21]. *F. columnare* or its DNA were not detected in these samples.

### Bacterial propagation

For each fish species, a highly virulent (HV) and a low virulent (LV) isolate with a known virulence profile, as described by Declercq et al., were used [16]. Isolates that were able to elicit 80% mortality or more within 72 h were assigned as HV, whereas isolates causing 20% mortality or less within the time-course of the 7 days experiment were designated LV [16]. Carp were experimentally inoculated with isolates 0901393 (HV) and CDI-A (LV), obtained from diseased carp. Rainbow trout were challenged by isolates P11/91 (HV) and JIP 44/87 (LV), sampled from diseased trout [20]. All four isolates belonged to genomovar I, as determined at the Aquatic Microbiology Laboratory of Auburn University (USA) using 16S-Restriction Fragment Length Polymorphism according to the protocol described by Olivares-Fuster et al. [22]. For more information concerning origin of the isolates, the reader is referred to Declercq et al. [20].

The isolates were grown for 36 h at 28 °C on modified Shieh agar plates [17,18]. For each isolate, five colonies per plate were sampled and transferred to 15 mL Falcon tubes filled with 4 mL of modified Shieh broth, which were placed overnight on a shaker at 28 °C at 100 rpm. The content of two Falcon tubes of these initial broth cultures was added to an additional 392 mL of modified Shieh broth in 500 mL glass bottles and again incubated for 24 h at 28 °C on a shaker at 100 rpm. The content of these bottles was used in the immersion challenge studies and the bacterial titres were determined by making tenfold dilution series in triplicate on modified Shieh agar plates.

### Experimental challenge

A group of 27 randomly chosen carp or rainbow trout was removed from the stocking tanks and placed in a 10 L inoculation tank filled with 4.6 L of aerated water at 27 ± 1 °C (carp) or 23 ± 1 °C (rainbow trout). Per fish species, three predetermined groups were included in duplicate; fish to be inoculated with either the HV- or LV-isolate and a control group. Then, 400 mL of cultivated modified Shieh broth containing the *F. columnare* isolates was added to the proper water tank. For the carp, the bacterial titres of the HV-isolate were 3.2 or 6.4 × 10^7^, for the LV-isolate 1.6 or 4.0 × 10^7^ CFU/mL. For the trout, the bacterial titres of the HV-isolate were 6.4 × 10^7^ or 1.6 × 10^8^ CFU/mL, and for the LV-isolate 3.2 or 6.4 × 10^7^ CFU/mL. A control group was included, constituting fish immersed in a tank with water supplemented with 400 mL modified Shieh broth not containing *F. columnare*. After a 90 min inoculation period, each group of 27 fish was transferred to a 60 L tank with trickling filter filled with 48 L of recirculated, aerated tapwater of 25 ± 1 °C for the carp or 22 ± 1 °C for the rainbow trout. The fish were clinically monitored every 30 min and three fish per tank were sacrificed with an overdose of benzocaine (10 g/100 mL acetone) at nine predetermined time-points i.e., 1, 2.5, 4, 6, 8, 9.5, 15.5, 24 and 36 h post inoculation (pi). As soon as the humane endpoints (isolation in a corner, swimming at the water surface, loss of balance) were reached, the fish were sacrificed with an overdose of benzocaine. In case dead fish were encountered, these were immediately removed from the aquaria and sampled only for bacteriological analysis, and not for histological nor ultrastructural examination. Of all sacrificed fish, the gill arches were inspected and the first two left gill arches were removed and sampled for histological, (immuno)histochemical, scanning (SEM) and transmission (TEM) electron microscopic examination. In all animals, the counterpart right gill arches served for bacteriological examination for *F. columnare* by means of bacterial titration by making tenfold dilution series of the gill tissue in triplicate on modified Shieh agar plates; additionally in the control animals PCR was performed as described before [16]. All experiments were approved by the Ethical Committee of the Faculty of Veterinary Medicine, Ghent University under the project number EC2012/60.

### Histological and (immuno)histochemical examination

Histological sections were used to enable a step-by-step tracking of microscopically discernible gill lesions in the course of time. Particular attention was paid to the localisation and arrangement of long slender bacterial cells with the typical *F. columnare* morphology, possible shifts in amount and type of mucins, and the type and spread of gill lesions (top, middle or base of the filaments and lamellae).

The gill tissue sections were fixed for 24 h in 4% phosphate-buffered formaldehyde, dehydrated in graded alcohol-xylene series and embedded in paraffin wax using the STP 420 Microm Tissue Processor and the embedding station EC 350–1 and 2 (Microm, Prosan, Merelbeke, Belgium), respectively. All tissues were sectioned (8 μm) (Microm microtome HM 360, Prosan) and stained with haematoxylin and eosin (H&E). A combined periodic acid Schiff/alcian blue (PAS/AB) stain at pH 2.5 was additionally applied, allowing mucous cells/mucin to stain blue (AB-positive, acid mucins), purple (PAS/AB-positive, neutral combined with acid mucins) or magenta (PAS-positive, neutral mucins).

In addition, (immuno)histochemistry was adopted to visualize apoptotic cells and ATP-ase activity of chloride cells. Therefore, 5 μm thin paraffin embedded tissue sections were mounted on 3-aminopropyl-triethoxysilane-coated slides (APES, Sigma, St. Louis, MO, USA), dried for 1 h at 60 °C on a hot plate and further dried overnight at 37 °C. To discern apoptosis, the terminal deoxynucleotidyl transferase (TdT)-mediated deoxyuridine triphosphate (dUTP) nick end-labelling (TUNEL) methodology was used for discerning DNA fragmentation. The TACS™ TdT in situ apoptosis detection kit (R&D Systems Europe Ltd, Abingdon, UK) was adopted following the protocol as described by Van Cruchten et al. [23]. In addition, caspase-3 activity was determined using a polyclonal rabbit IgG human/mouse active caspase-3 antibody (1/400, R&D Systems Europe Ltd, Abingdon, UK) and the Anti-Rabbit HRP-AEC Cell & Tissue Staining Kit (R&D Systems Europe Ltd, Abingdon, UK). The protocol employed was modified from Van Cruchten et al. [23] with the difference that 50 μL of a labelled polymer of the Dako EnVision + System/HRP, Rabbit (DAB+) kit was used according to the instructions of the manufacturer. To detect the ATPase activity of the chloride cells, a monoclonal mouse antibody Na, K- ATPase (1/200, University of Iowa, Department of Biological Sciences) and the Dako EnVision + System/HRP, Mouse (DAB+) (DakoCytomation, Glostrüp, Denmark) staining kit (Ref.K4007) were applied according to the instructions of the manufacturer.

The (immuno)reactive cells on the (immuno)histochemically stained sections and the PAS-positive, PAS/AB-positive, AB-positive goblet cells and eosinophilic granular cells (EGC) on PAS/AB-stained sections were quantified on three randomly selected gill filaments. Counting was done at the tip, middle and base of these filaments. For all goblet, EGC- and chloride cell counts, results are expressed as the number of cells per 100 μm gill filament. For the TUNEL- and caspase-3-techniques, the results are presented as the number of positive cells per 1000 μm lamellar surface. Apoptosis and chloride cell activity were only determined at the first four sampling points.

### Electron microscopy

For SEM, the gill samples were preserved in a HEPES-glutaraldehyde solution. Tissue samples were postfixed in 1% buffered osmium tetroxide for 2 h and dehydrated in an increasing alcohol series followed by increasing ethanol–acetone series up to 100% acetone. The samples were then dried to the critical point with a Balzers CPD 030 critical point drier (Sercolab bvba, Merksem, Belgium) and further mounted on metal bases and sputtered with platinum using the JEOL JFC 1300 Auto Fine Coater (Jeol Ltd, Zaventem, Belgium). The samples were examined with a JEOL JSM 5600 LV scanning electron microscope (Jeol Ltd). For TEM processing, a protocol as described by De Spiegelaere et al. [24] was used. For examination of the TEM-samples, a JEM-1400 plus Jeol electron microscope (Jeol Ltd) operating at 80 kV was used. Micrographs were taken digitally.

### Statistical analysis

The effect of three independent variables (degree of virulence of bacterial strain, time point after inoculation and localization on the gill filament) on nine dependent variables (number of recovered bacteria, presence of chloride cells, EGC (only in carp), PAS-positive, PAS/AB-positive, AB-positive, total goblet, TUNEL- and caspase-3 positive cell counts), was assessed. Since a clearly distinct result was noted in some carps inoculated with the LV-isolate in terms of mortality and macroscopically discernible gill lesions, this group was further subdivided into a group of fish displaying no macroscopic abnormalities (further denoted as the carp inoculated with the LV-isolate) and a group of fish that died with grossly visible gill lesions comparable to the macroscopic lesions as seen in fish exposed to the HV-isolate (further denoted as the LV-isolate affected fish).

As the bacterial titration counts for carp and rainbow trout were not normally distributed, the data were log transformed for further statistical analysis.

Depending on the distribution of the dependent variables, two different statistical assays were performed. Firstly, the effect of the independent variables on the outcome of bacterial titrations, PAS/AB-positive goblet cells, total goblet cells, chloride cells, TUNEL and caspase-3 positive cells in carp, and chloride cells, TUNEL, and caspase-3 positive cells in rainbow trout were assessed using a multivariate linear mixed model. When a significant effect on one of the independent variables was observed in the multivariate model, post hoc comparisons were performed using Scheffe or least significant difference (LSD) tests.

Secondly, the data of PAS-positive goblet cells, AB-positive goblet cells and EGC in carp, and PAS-positive goblet cells, PAS/AB-positive goblet cells, AB-positive goblet cells and total gill goblet cells in rainbow trout, were transformed into a binary dataset with values “absence of cells (=0)” and “presence of cells (=1)” and analysed by means of a multivariate logistic regression model.

Statistical results were considered to be significant when *p*-values were below 0.05. All analyses were performed using SPSS version 21.0.

## Results

At the last two sampling points (SPs) i.e. 24 h and 36 h pi, no fish in the groups challenged with the HV-isolates were remaining. Therefore, these SPs were omitted. As the disease progressed markedly faster in trout challenged with the HV-isolate from SP5 onwards, the remaining trout of all groups were sacrificed 3.5 h earlier compared to the carp hence advancing the last SP to 12 h instead of 15.5 h pi.

The control animals of both carp and rainbow trout remained clinically healthy throughout the experiment and no mortality occurred. No lesions nor *F. columnare* bacterial cells were observed upon macroscopic, light microscopic and ultrastructural examination of the gill tissue. Additional light microscopic images display the normal gill histology of a carp (Additional files 1 and 2). Additional scanning electron microscopic images demonstrate the intact gill filaments and gill lamellae in the control animals (Additional files 3 and 4). Skin lesions were not observed in any of the animals during the trials.

## Carp

### Chronological changes in type and extent of gill lesions

#### Following challenge with the HV-isolate

##### SP 1 and 2 (1 and 2.5 h pi, respectively)

No macroscopic lesions were noted and none of the 12 fish sampled or any of the fish present in the tank displayed any clinical signs of discomfort or disease. No dead fish were encountered.

**Figure 1:**
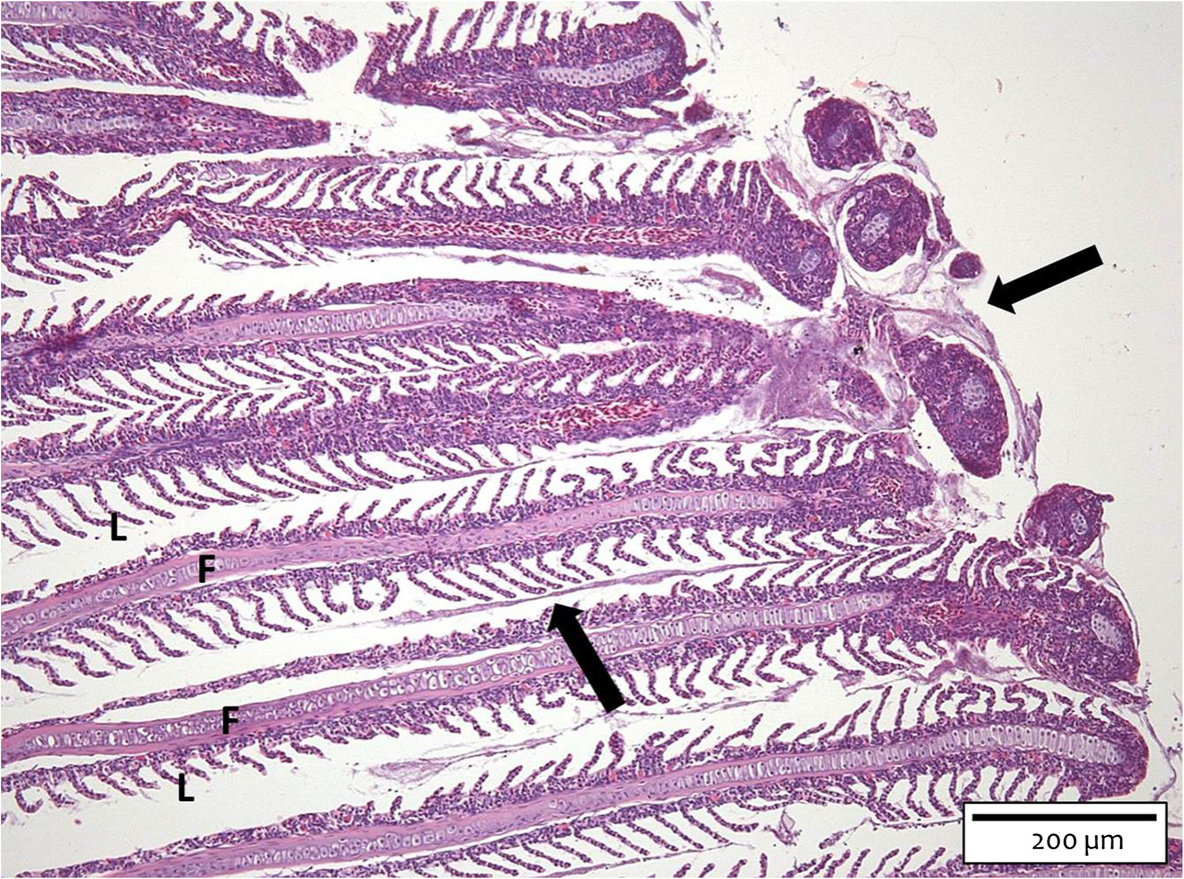
**Gill section of a carp challenged with the HV-isolate at SP 2.** The bacterial cells (arrows) are clustered focally in an eosinophilic matrix encompassing the tips of the gill filaments (F) and extending to the middle of the gill filaments. L = gill lamella (H&E, bar = 200 μm).

Histological imaging revealed bacteria clustered focally in an eosinophilic matrix encompassing the tips of one to three (first SP) and two to six (second SP) out of the six discernible filaments. The lamellae that were in the vicinity of these bacterial clusters were oedematous at the first SP. The lamellae visualized on the sections of the second SP revealed fusion and necrosis. In these necrotic areas, the bacteria were closely associated with the denuded lamellar epithelium with only pillar cells still showing an intact structure. In addition, the bacterial cells had further migrated to the middle of the filaments (Figure 1). Filamental architecture was safeguarded in all fish examined.

**Figure 2:**
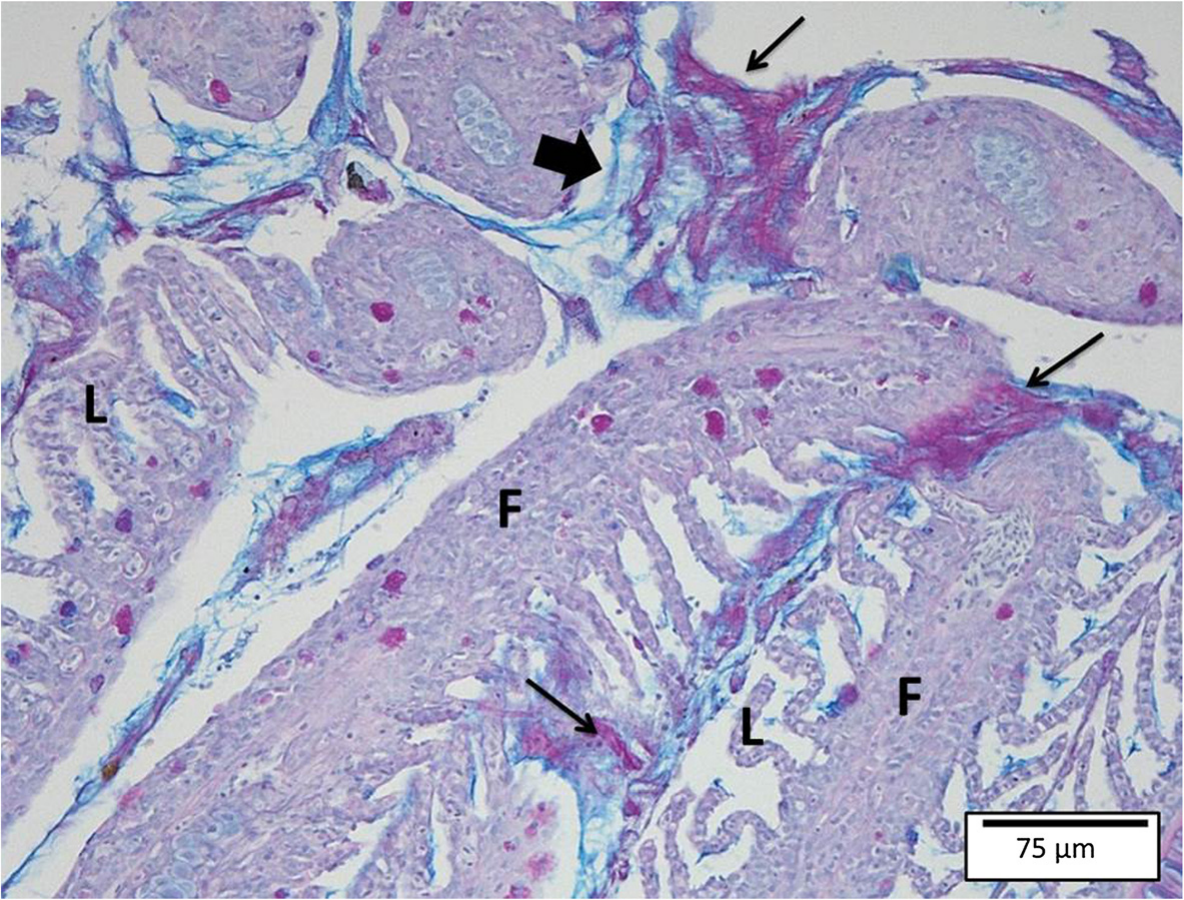
**Gill section of a carp challenged with the HV-isolate at SP 2.** The bacterial cells are surrounded by a PAS-positive matrix (thin arrows, magenta) enveloped by AB-positive mucins (thick arrow, blue). F = gill filament; L = gill lamella (PAS/AB, bar = 75 μm).

PAS-staining showed a PAS-positive matrix immediately surrounding the bacteria that in turn was enclosed by AB-positive mucins (Figure 2). Both layers exhibited the same thickness of 3-30 μm, with the thickness of the surrounding layer increasing with the size of the bacterial cluster.

**Figure 3:**
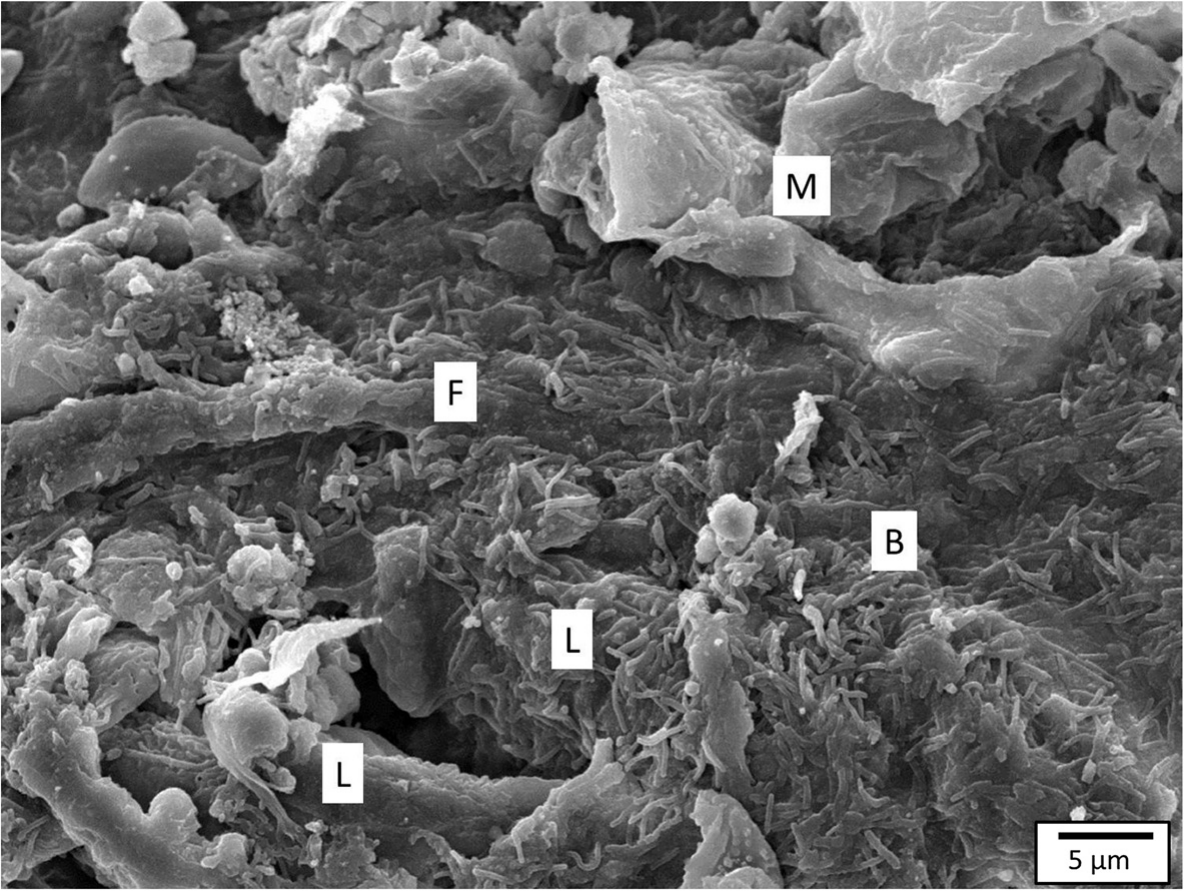
**Carp gill after inoculation with HV-isolate at SP 2.** The gill filament (F) and gill lamellae (L) are covered by long, slender bacterial cells (B), clustering in between mucus (M) and cell debris (SEM, bar = 5 μm).

SEM (Figure 3) and TEM revealed aggregates of long, slender bacterial cells, with large clumps of these bacterial cells encompassing the filament tips at the second SP.

##### SP 3 and 4 (4 h and 6 h pi, respectively)

At 6 h pi, the first moribund fish appeared. These two fish were hanging at the surface or in a corner of the tanks, displayed loss of balance and were gasping for air. These two (out of the six) sampled fish displayed macroscopic lesions. The latter consisted of foci of whitish discolouration of the first gill arch on both sides. One dead fish was encountered.

Upon inspection of the histological sections from the six fish sacrificed at 4 h pi, assembled bacterial cells were noted in close proximity to the lamellar epithelium of the filament tips with focal loss of filamental architecture. In addition, bacterial cells had pursued their way to the filament base. The gill tissue of the fish sampled at 6 h pi displayed multiple bacterial micro-colonies smothering half to all filament tips coinciding with lamellar fusion and filament destruction.

**Figure 4:**
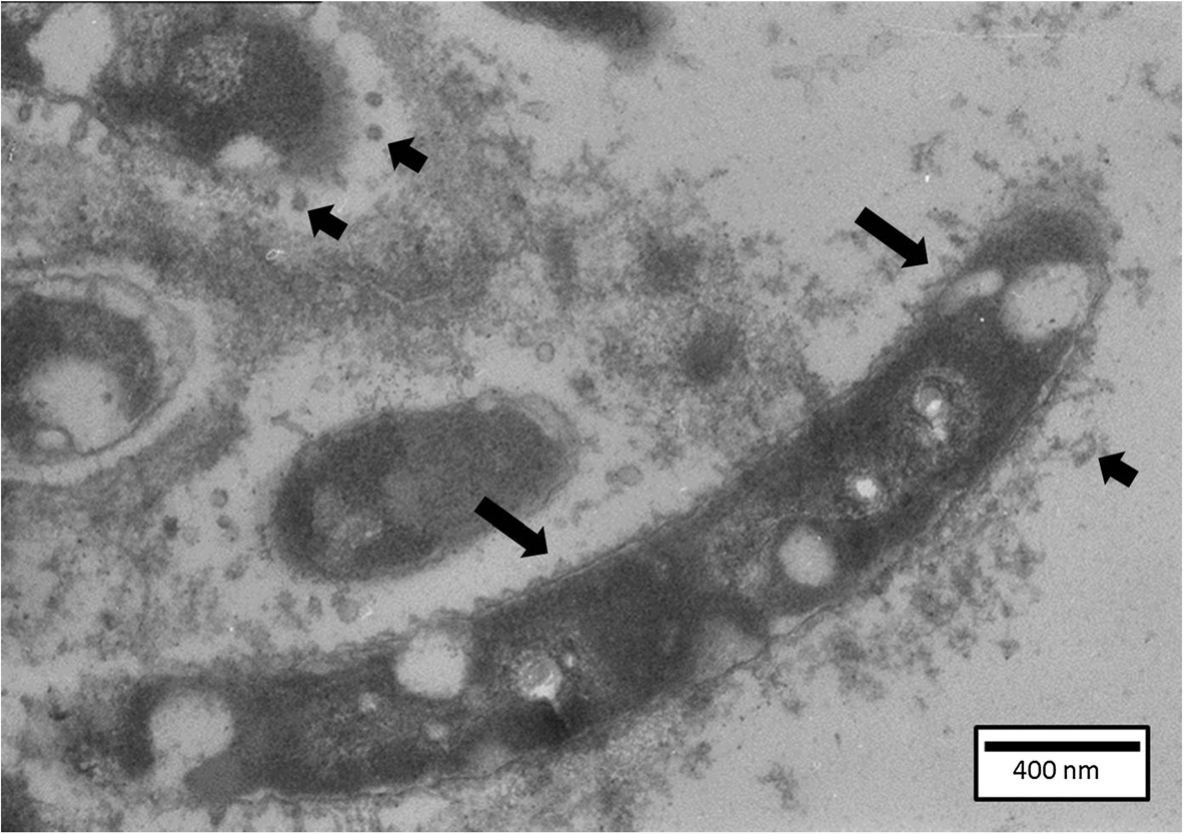
**Carp gill after inoculation with the HV-isolate at SP 3.** Bacterial cells present a knurled outer membrane (long arrows), and are regularly surrounded by outer membrane vesicles (short arrows) (TEM, bar = 400 nm).

SEM confirmed the presence of huge clusters of densely packed bacterial cells wrapped in cellular debris and covering the gill tissue (Additional file 5). TEM-examination revealed bacterial cells directed parallel to and in intimate contact with the gill epithelia. In conjunction with bacterial presence, severe gill damage with oedema and cell necrosis was noted. The outer membrane of the bacteria was remarkably knurled and regularly surrounded by outer membrane vesicles (OMV) (Figure 4).

##### SP 5, 6 and 7 (8, 9.5 and 15.5 h pi, respectively)

The vast majority of fish displayed overt signs of disease as exhibited by loss of equilibrium, isolation and respiratory distress, with an increase in severity at later SPs. All these fish showed macroscopic lesions, as manifested by multifocal discolouration of the first gill arches bilaterally. Thirteen dead fish were encountered. Two out of 18 sampled fish remained clinically healthy with no apparent lesions. At the last SP, only four fish (two out of each aquarium) were remaining and sampled for (immuno)histochemical, ultrastructural and bacteriologic analysis.

**Figure 5:**
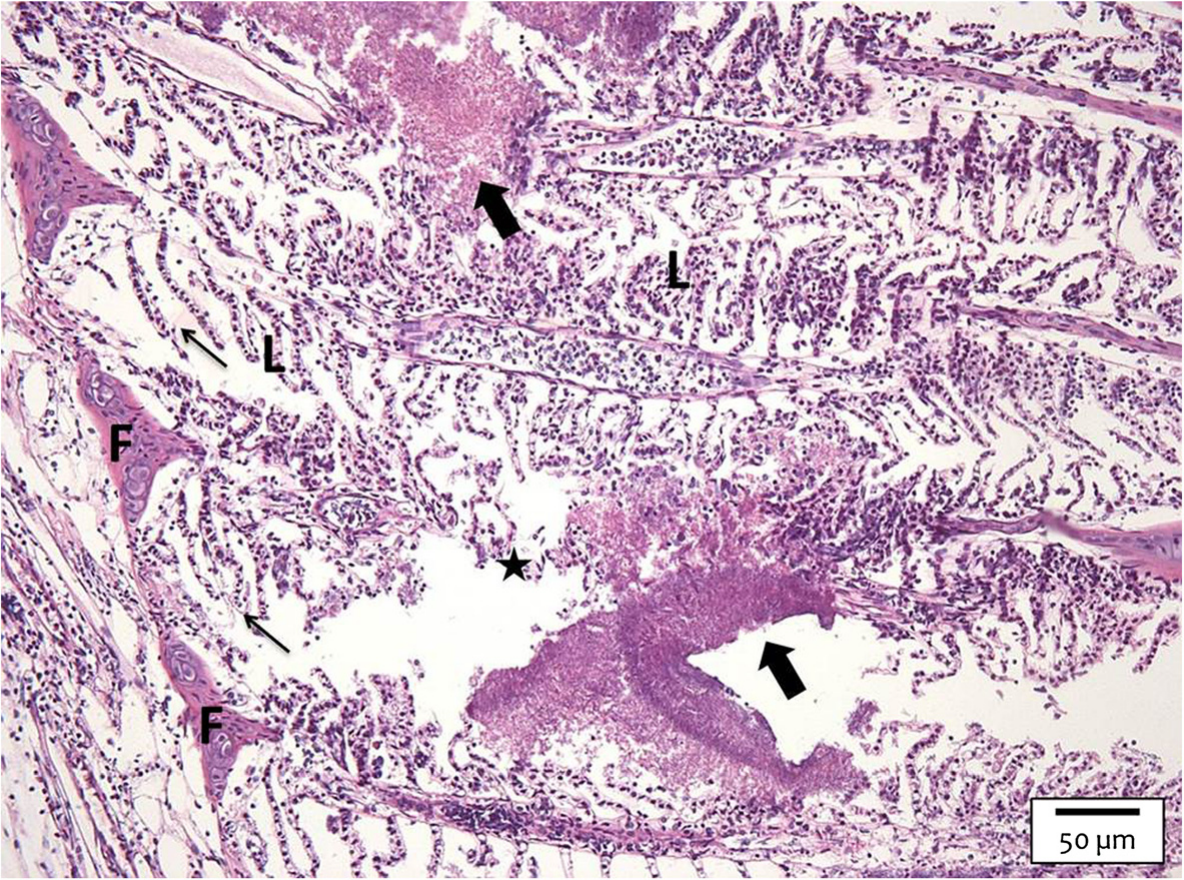
**Gill section of a carp challenged with the HV-isolate at SP 7.** Large clumps of bacterial micro-colonies (thick arrows) embedded in an eosinophilic matrix are discerned. Areas of complete architectural loss (asterisk) are noted in close proximity of bacterial clumps. Overall oedema (thin arrows) and total lamellar (L) and filament (F) fusion are apparent (H&E, bar = 50 μm).

The histological image changed into severe pathological lesions in all fish sampled, except for the two clinically healthy animals. Large clumps of bacterial micro-colonies, embedded in an eosinophilic matrix, covered all filament tips. Multifocally spread bacterial cells were seen in close contact with the lamellar epithelium over the full length of the gill filaments. Focal lamellar fusion and tissue necrosis were associated with the bacterial cells with significantly more fusion of the lamellae at the filament tips compared to the control animals. Areas of complete architectural loss in close proximity of bacterial clumps were additionally noted. At SP 7, overall oedema and total lamellar and filamental fusion were apparent (Figure 5).

TEM revealed all bacterial outer membranes consistently surrounded by OMV. SEM confirmed the histological findings and was demonstrated by large clumps of bacterial micro-colonies (Additional file 6), embedded in a matrix, covering all filament tips.

#### Following challenge with the LV-isolate

##### SP 1 and 2 (1 and 2.5 h pi, respectively)

No clinical signs of discomfort nor macroscopic lesions were seen. No mortality was discerned.

Histological examination revealed the presence of clusters of bacterial cells in close contact with one to three (SP 1) and two to three (SP 2) out of six filament tips in all 12 fish sampled. These bacterial aggregates were also found in the middle of the filaments. The gill filament and lamellae did not exhibit any abnormalities, except for focal lamellar oedema in the filament tips in the presence of bacterial cells.

PAS-staining showed a PAS-positive matrix immediately surrounding the bacteria that in turn was enclosed by AB-positive mucins with the larger the bacterial cluster, the thicker the surrounding layers.

**Figure 6:**
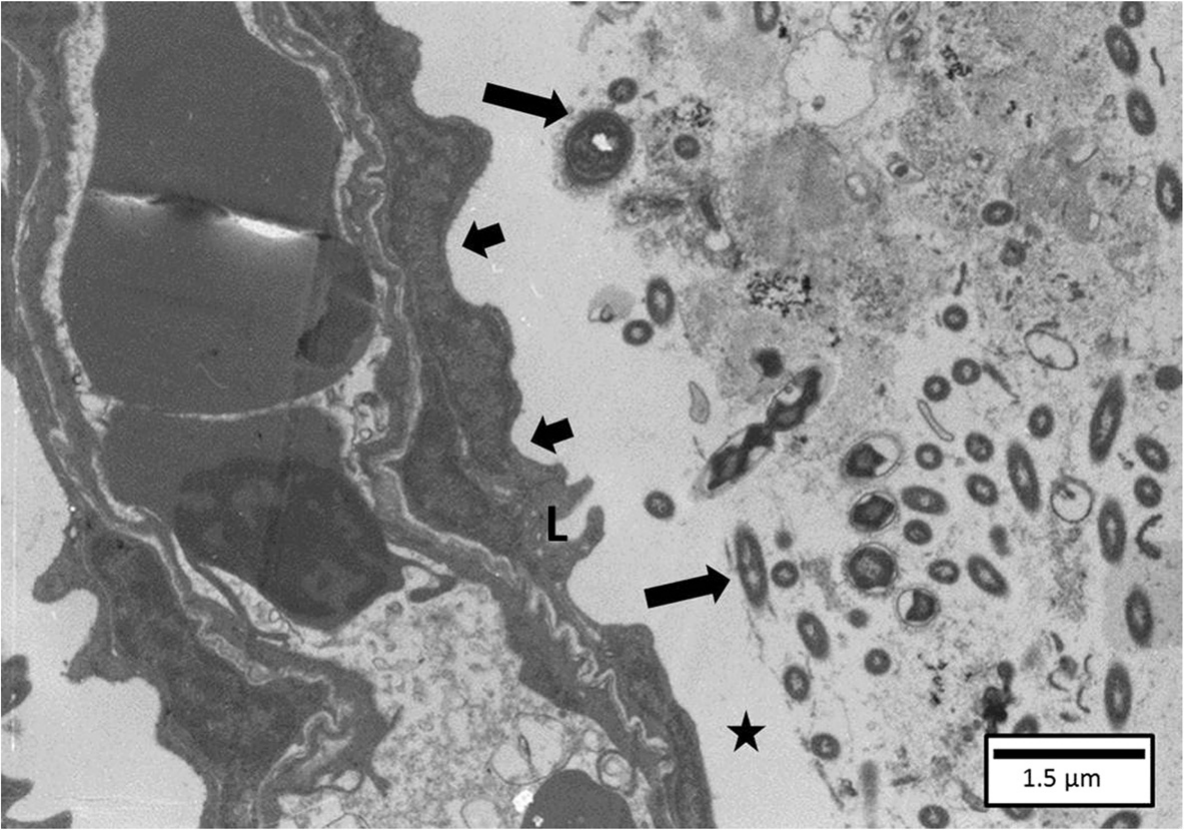
**Gill of a carp inoculated with the LV-isolate at SP 2.** A cluster of bacterial cells (long arrows) is separated from the lamellar epithelium (L) (short arrows) by a translucent layer (asterisk) (TEM, bar = 1.5 μm).

TEM examination revealed bacterial cells that appeared to be separated from the epithelium by a translucent layer (Figure 6). In one fish sampled at 2.5 h pi, close contact between the bacterial cell and the gill epithelium was noted, with a parallel orientation of the bacterium towards the lamellar epithelial cells and apparent bacterial cell division. The bacterium showed a cell wall with an apparently less knurled outer membrane compared to the bacterial cells of the HV-isolate. OMV were observed only in a minority of the bacterial cells. Bacteria could not be visualized using SEM examination. Indeed, only huge mucus clots and packed cells were evident mostly at the gill filaments tips, covering the gill tissue. In sites not covered by mucus, the normal fish gill fingerprinting pattern was visible.

##### SP 3 and 4 (4 and 6 h pi, respectively)

Two fish out of twelve sampled exhibited severe signs of discomfort and were swimming at the surface, gasping for air. However, no macroscopic lesions were encountered in any of the fish sampled. No other clinical abnormalities nor dead fish were observed.

Histological examination revealed the presence of bacterial cells in nine out of the twelve fish sampled. Bacteria forming small micro-colonies surrounded by an eosinophilic matrix and necrotic cells were observed in close contact with the gill epithelium at the tips and middle of the filaments. The two clinically affected fish showed multifocal histological filament destruction as seen in the fish inoculated with the HV-isolate. No abnormalities were discerned in the outnumbering clinically healthy fish that were sacrificed.

SEM and TEM endorsed the histological results with bacterial cells being noted enclosed in mucus clots and in close contact with the gill epithelium, respectively. As in the HV-isolate, the outer membrane of the bacterial cell wall was surrounded by OMV, but only a minority of the bacterial cells revealed this phenomenon. Again, a less knurled outer membrane was noted.

##### SP 5, 6 and 7 (8, 9.5 and 15.5 h pi, respectively)

Three fish were moribund at SP 6 and 7 and revealed macroscopic lesions consisting of multifocal whitish discolouration of the first gill arches. One dead fish was encountered.

The gills of the clinically healthy fish did not reveal any macroscopic or microscopic abnormalities and no bacterial cells were noted. The histological findings of the gills of the three moribund fish were comparable to those as described in the fish inoculated with the HV-isolate in terms of the elicited lesions and spread of the bacterial cells throughout the gill tissue. Indeed, huge clusters of bacteria embedded in an eosinophilic matrix with necrotic debris were sited at the filament tips, with offshoots of these in close contact with the gill epithelium over the entire length of the gill filaments. In these sites, severe oedema, lamellar fusion and lamellar gill necrosis were present. Significantly more fusion of the lamellae was observed in the gills, especially at the filament tips, of fish inoculated with LV-isolate as compared to the control animals. At no time, the cartilaginous tissue was lysed nor were the gill filaments fused.

SEM and TEM of the gills of the moribund fish showed bacterial cells wrapped in a matrix of cells and mucus and contact with epithelial cells, respectively. No abnormalities in the clinically healthy fish were encountered.

### Chronological changes in apoptotic, eosinophilic granular, goblet and chloride cells

With regard to the first four SPs, a borderline non-significant main effect of group for TUNEL-positive cells was observed (*p* = 0.07), but the post hoc tests (LSD) showed that the mean difference between the TUNEL-positive cell count per 1000 μm gill filament contour in the gills of carp challenged with the HV-isolate compared to the control animals was 1.65 ± 0.64 (*p* < 0.05). No other significant differences were noted for the TUNEL-positive cell counts. The main effect of group for caspase-3 immunoreactive cell counts per 1000 μm gill filament contour was highly significant (*p* < 0.001). The post hoc tests (LSD) revealed that the mean difference between the caspase-3 immunoreactive cell counts per 1000 μm gill filament contour in the gills of carp inoculated with the HV-isolate and the control animals was 2.3 ± 0.5 (*p* < 0.001); a comparable difference was noted between gill tissue of carp challenged with the LV-isolate and the control animals (*p* < 0.001). Most caspase-3-immunoreactive cells were noted at the second SP, followed by a decreasing trend. The vast majority of cells staining positive with either TUNEL or caspase-3 were epithelial cells, with only occasionally a positive goblet cell discerned. At no time, significant differences between the various groups were noted for the number of chloride cells per 100 μm gill filament length. Lysis of these cells was found, especially when oedema was present. The gills of fish exposed to the HV-or LV-isolate displayed lysis of chloride cells, although to a higher degree in the HV-isolate inoculated fish which showed more oedema.

**Figure 7:**
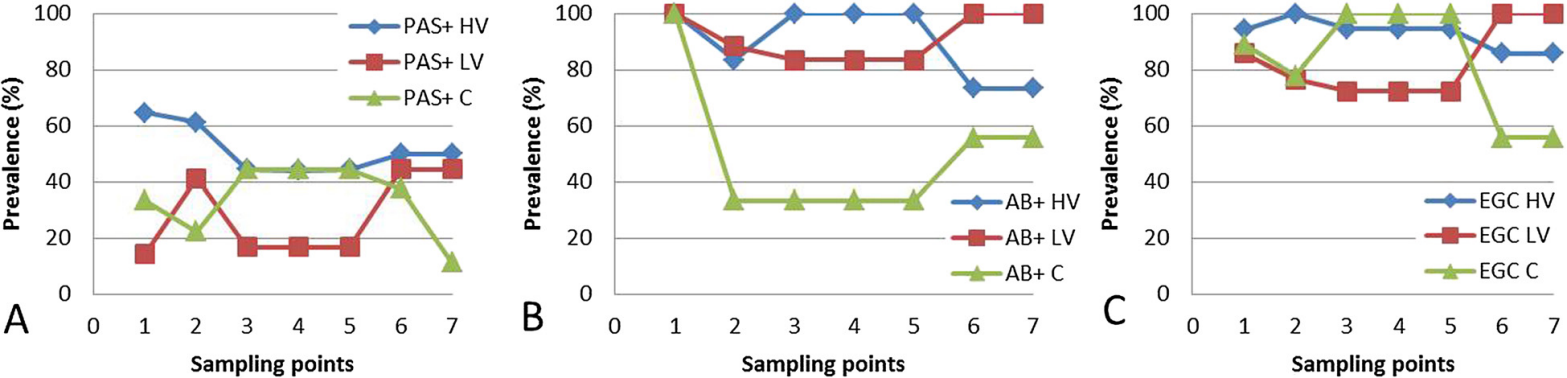
**Prevalence (in%) of PAS-positive (A), AB-positive (B) and EG (C) cells in carp.** The values are calculated per sampling point, for fish belonging to a certain virulence group sampled. Significantly higher odds were found for PAS-positive and AB-positive cells counted in the gills after inoculation with the HV-isolate (blue) compared to the control animals, and for the AB-positive cells in the gill tissue of the LV-isolate (red) challenged fish compared to the control animals (green). Significantly higher odds were encountered for the EGC counted in the gills of fish challenged with the HV-isolate compared to the control fish, and for the HV-isolate compared to the LV-isolate inoculated fish.

The main effect of group for the total gill goblet cell counts was significant (*p* < 0.01). In the post hoc test (Scheffe), the mean difference between the total gill goblet cell count per 100 μm gill filament length of fish inoculated with the HV-isolate and the control animals was 0.83 ± 0.27 (*p* < 0.05), while the mean difference between fish inoculated with the LV-isolate and the control animals was 1.06 ± 0.31 per 100 μm gill filament length (*p* < 0.01), with no other significantly different results occurring in total goblet cell counts. For the PAS/AB-positive cell counts, no significant differences occurred between any of the groups. The main effect of group for PAS- and AB-positive cell counts was significant (*p* < 0.01). Upon comparing the cell counts in the gill tissue after HV-isolate inoculation with the control group, data revealed that the former had a 2.86 and 4.76 times higher odd (*p* < 0.01) for PAS-positive and AB-positive cell counts per 100 μm gill filament length, respectively. Higher counts were noted in the vicinity of bacterial cells, mostly at the tips of the gill filaments. Moreover, 3.85 times higher odds (*p* < 0.01) for the AB-positive cells per 100 μm gill filament length were encountered in the gills after challenge with the LV-isolate compared to the control animals and even 5.22 times higher odds (*p* < 0.01) were found for the AB-positive cells in the gills of LV-isolate affected fish compared to the control fish. The main effect of group for EGC positive cell counts was significant (*p* < 0.05). Upon comparing the cell counts in the gill tissue after inoculation with the HV-isolate with the control group, data revealed that the former had a 4.44 times higher odd per 100 μm gill filament length (*p* < 0.01). Data revealed that the EGC counts of control fish, fish inoculated with the LV-isolate and LV-isolate affected fish had a 0.23, 0.32 and 0.24 times lower odd (*p* < 0.05), respectively, upon comparing the cell counts in the gill tissue after HV-isolate inoculation. No other significant differences were found in the EGC count. In gill sections of fish exposed to the HV-isolate, upon inspection of sites of tissue damage, degranulation of EGC was noted along with their migration onto the gill lamellae. A dynamic overview of PAS-positive goblet cells, AB-positive goblet cells and eosinophilic granular cells in carp is presented in Figure 7.

### Temporal changes in bacterial cell counts

**Figure 8:**
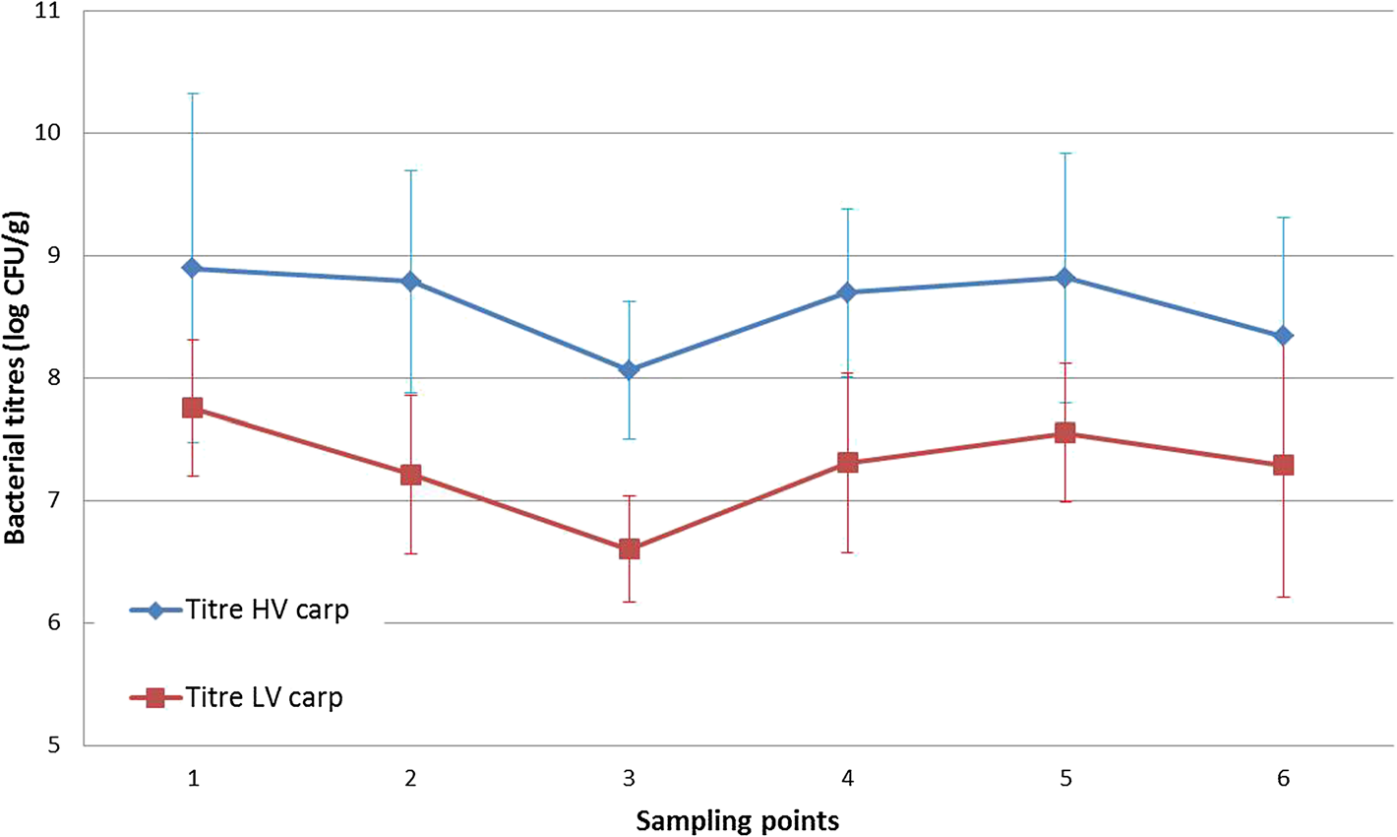
**Gill bacterial titres (log CFU/g) after challenge with the HV- and LV-isolate in carp.** The bacterial titres of fish inoculated with the HV-isolate (blue) remain high during the course of the experiments and show a significant higher difference compared to the fish infected with the LV-isolate (red). The bacterial titres retrieved from the latter carp follow a same trend compared to the HV-isolate and remain high until the end of the experiment.

The bacterial titres retrieved from the gill tissue at the first SP were the highest during the course of the experiment (7.8 × 10^8^ CFU/g gill tissue and 5.6 × 10^7^ CFU/g gill tissue for the fish inoculated with the HV- and LV-isolate, respectively). A tenfold decrease was noted towards the third SP, after which titres increased again approaching the values of the first SP. Subsequently, the bacterial titres either stagnated or decreased gradually. An overview of the average bacterial titres retrieved from the gill tissue after exposure with the HV- and LV-isolates can be found in Figure 8. Overall, the mean bacterial logarithmic titres retrieved from gill tissue in the HV- and LV-challenged fish were ^10^log 7.92 ± 0.15 CFU/g and ^10^log 6.70 ± 0.18 CFU/g, respectively. The LV-isolate affected fish had a mean bacterial logarithmic titre of ^10^log 7.15 ± 0.39 CFU/g. There was a clear significant main effect of group in the bacterial titres (*p* < 0.001). In the post hoc tests (Scheffe), the mean difference for the bacterial cell counts between the HV- and the LV-isolate challenged fish and the LV-isolate affected fish was ^10^log 1.15 ± 0.21 (*p* < 0.001) and ^10^log 1.10 ± 0.38 (*p* < 0.05) CFU/g, respectively. No *F. columnare* cells were detected in the gill tissue of the control animals.

## Rainbow trout

### Chronological changes in type and extensiveness of gill lesions

#### Following challenge with the HV-isolate

##### SP 1 and 2 (1 and 2.5 h pi, respectively)

No clinical signs of discomfort nor macroscopic lesions were noted in any of the 12 sampled fish or any of the other fish present in the tanks. Three dead fish were encountered revealing no macroscopic abnormalities.

Histologically, the first lesions were noted as mild oedema and hyperplasia in six out of twelve sampled fish. The other half of the fish showed focal to generalized severe oedema with detachment of the epithelium from the underlying intact pillar cells. Bacterial cells were spotted focally, mostly encompassing the filament tips and middle of the gill filaments. Their presence was associated with localized lamellar necrosis, while the neighbouring lamellae remained intact, apart from slight oedema of the epithelium on the colonized side.

**Figure 9:**
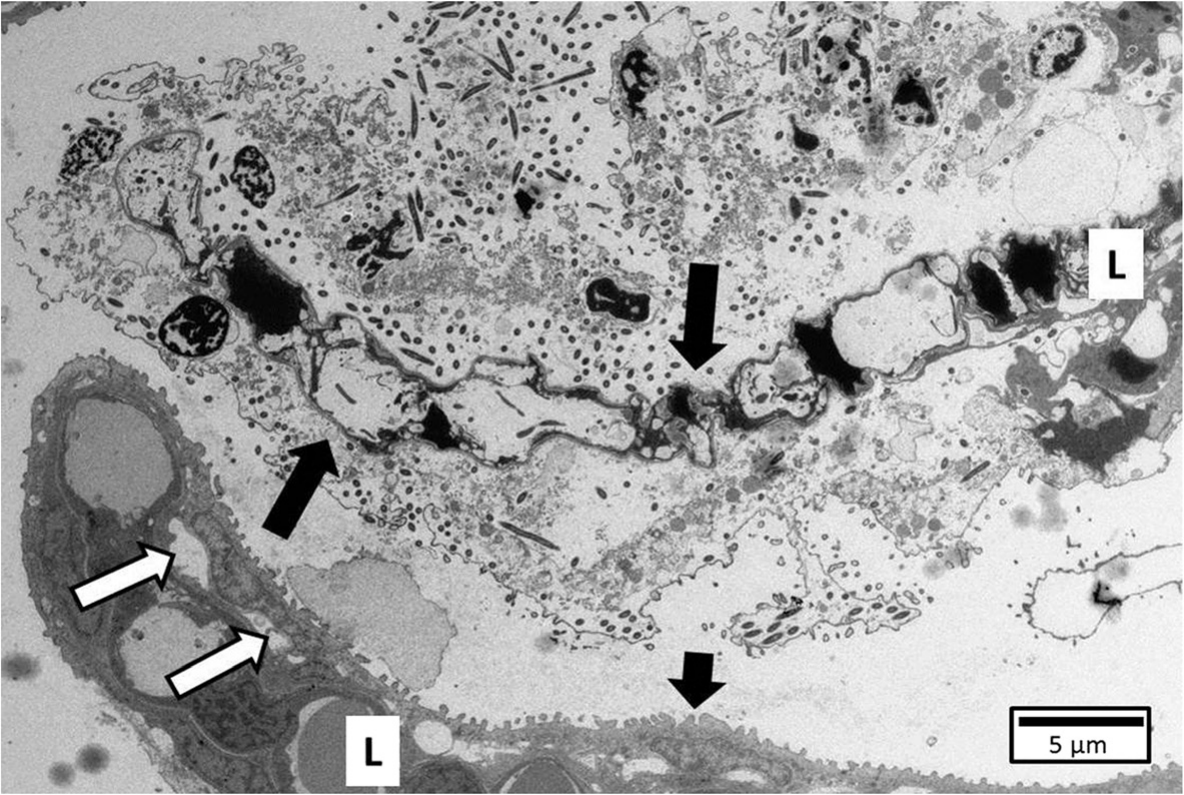
**Gill tissue of a trout following exposure to HV-isolate at SP 1.** Localized necrosis (long arrows) is observed in a gill lamella (L) while the neighbouring lamella remains intact (short arrow), apart from slight oedema (white arrows) of the epithelium on the sides where bacteria are present (TEM, bar = 10 μm).

TEM confirmed the latter finding (Figure 9). Furthermore, TEM revealed the chromatin of the nucleus to be marginalized and clumped while the other cell organelles remained intact, as seen in apoptotic cells. SEM did not reveal abnormalities.

##### SP 3 and 4 (4 and 6 h pi, respectively)

Clinical signs of discomfort and macroscopic lesions were absent in all fish. One dead fish occurred, showing no macroscopic lesions.

Histologically, bacterial cells were noted in six fish out of twelve fish sampled. Small clumps of bacterial cells were found very focally over the entire length of the filaments and in close contact with the epithelium. Their presence coincided with severe oedema, lamellar necrosis and sloughing of epithelial cells.

PAS-staining revealed the bacterial clusters to be wrapped in a PAS-positive matrix while being encased in AB-positive mucus.

The histological findings were confirmed by SEM- and TEM-pictures revealing bacterial clumps present at the gill filament tips and in close contact with the epithelium, respectively.

##### SP 5, 6 and 7 (8, 9.5 and 12 h pi, respectively)

From SP 5 onwards, most remaining rainbow trout exhibited severe discomfort including an altered swimming pattern and gasping for air, and macroscopic focal whitish discolouration of the gill tissue. Eight dead fish were discerned. Only three fish out of the remaining 18 did not exhibit clinical nor macroscopic abnormalities the last three SPs.

Apart from the latter three fish mentioned, histological analysis showed small bacterial clumps in intimate contact with the gill epithelium over the entire length of the filaments, and affecting almost all lamellae. Consistent with the bacterial presence, severe, generalized oedema and lamellar necrosis occurred with shedding of the epithelial cells causing the underlying pillar cells to be denuded. The cartilaginous core of the filaments remained intact.

Likewise, SEM revealed bacterial clusters covering the gill epithelium as blankets and wrapped in between necrotic cells and cellular debris. TEM revealed a knurled outer membrane in the majority of bacterial cells with the consistent occurrence of OMV.

#### Following challenge with the LV-isolate

Neither clinical signs nor macroscopic lesions were seen in rainbow trout challenged with the LV-isolate during the entire experiment. No dead fish were encountered.

At the first three SPs, 14 out of the 18 sampled fish showed mild gill oedema, either focal or generalized, associated with hypertrophia of the epithelial cells situated at the basis of the lamellae. At the last four SPs, two out of 24 fish showed mild oedema with no further abnormalities. Bacterial cells were never discerned in any sample. SEM and TEM showed normal gill tissue with no bacteria present.

### Chronological changes in apoptotic, eosinophilic granular, goblet and chloride cells

With regard to the first four SPs, the main effect of group for TUNEL-positive cell counts was significant (*p* < 0.05). The post hoc tests (Scheffe) revealed that the mean difference between the TUNEL cell count per 1000 μm gill filament contour in the gills of fish challenged with the HV-isolate compared to the control animals was 2.67 ± 1.08 (*p* < 0.05). No other significant differences were noted for the TUNEL-positive cell counts. The main effect of place for TUNEL-positive cells was significant (*p* < 0.01). Post hoc results (Scheffe) showed that most TUNEL-positive cells per 1000 μm gill filament contour occurred at the filament tips with a mean difference of 3.30 ± 0.98 (*p* < 0.01) TUNEL-positive cells compared to the middle parts of the gill filaments. The main effect of group in caspase-3 immunoreactive cell counts per 1000 μm gill filament contour was significant (*p* < 0.01). The post hoc tests (Scheffe) revealed that the mean difference between the caspase-3 immunoreactive cell counts per 1000 μm gill filament contour of the gills of rainbow trout challenged with the HV-isolate and the LV-isolate inoculated fish was 2.02 ± 0.54 (*p* < 0.01) while between the HV-isolate inoculated fish and the control animals, this difference was 1.91 ± 0.68 (*p* < 0.05) per 1000 μm gill filament contour. No other significant differences occurred. Cells staining positive with either TUNEL or caspase-3 were predominantly epithelial cells, including occasional goblet cells. No significant differences occurred for the number of chloride cells per 100 μm filament length in between the various groups. As described for the carp, fish exposed to the HV- or LV-isolate displayed lysis of chloride cells but, as more oedema was noted following challenge with the HV-isolate, markedly more lysis of chloride cells was perceived in the fish exposed to the latter isolate.

As for the goblet cell count, no significant differences were noted in between any of the goblet cell counts in between the various groups. An increase in AB-positive cell count was noted in the HV-group from the second to the third SP though.

EGC were not observed in the gill sections of rainbow trout.

### Temporal changes in bacterial cell counts

**Figure 10:**
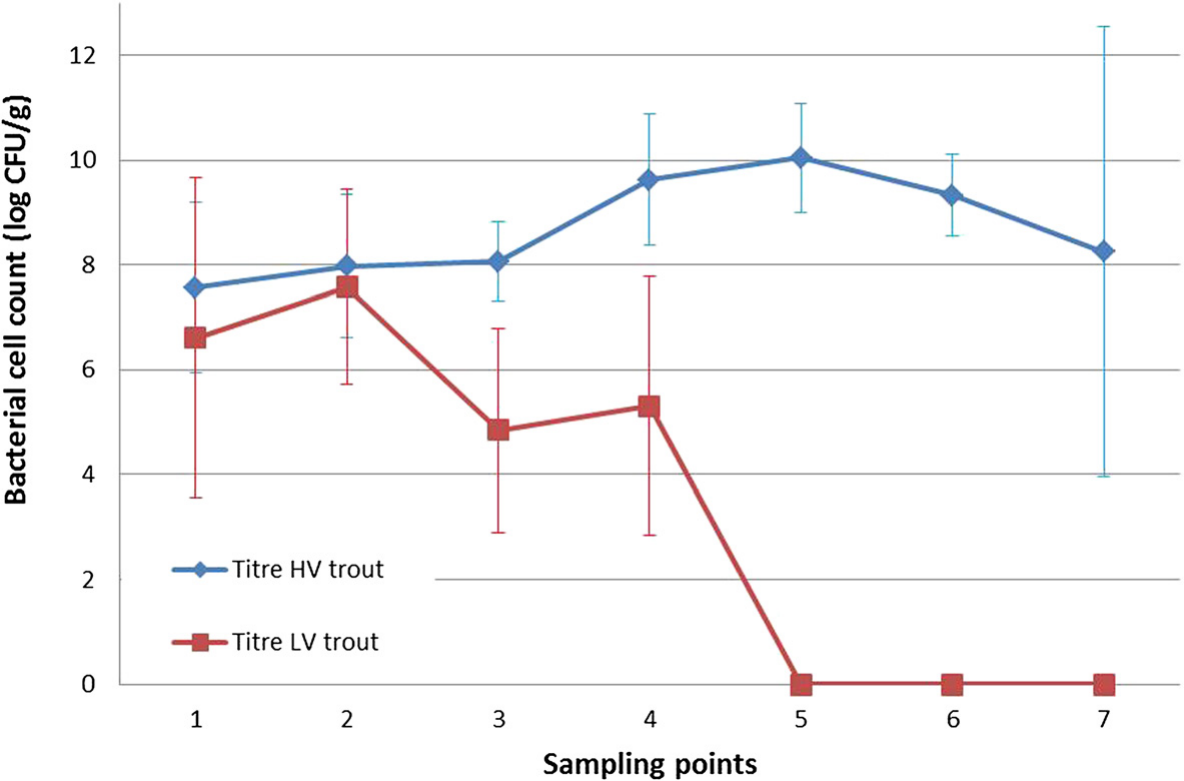
**Gill bacterial titres (log CFU/g) after challenge with the HV- and LV-isolate in trout.** The bacterial titres of fish inoculated with the HV-isolate (blue) remain high during the course of the experiments. The bacterial titres retrieved from fish infected with the LV-isolate (red) show progressively a substantially decreasing trend.

Bacterial titres from the gill tissue inoculated with the HV- and LV-isolate were ^10^log 8.03 ± 0.27 CFU and ^10^log 2.76 ± 0.42 CFU per g gill tissue, respectively. There was a clear significant main effect of group in the bacterial titres (*p* < 0.001). In the post hoc tests (Scheffe), the mean difference for the bacterial cell counts in between the HV-isolate challenged fish and the LV-isolate inoculated fish was ^10^log 5.26 ± 0.39 (*p* < 0.001) CFU/g. An overview of the bacterial titres retrieved from the gill tissue at the various SPs after challenge with the HV- and LV-isolate can be found in Figure 10. No *F. columnare* bacteria were retrieved from the control animals.

## Discussion

The purpose of this study was to track the evolution and discern conspicuous features of the gill lesions in experimentally induced columnaris disease. This is the first description of the sequence of events taking place at the level of the gill tissue before the fish succumb to columnaris disease.

In carp, as soon as 1 h post challenge, bacterial cells of the HV-isolate were found in close contact with the epithelium of the filament tips. Only 1 h later, attachment was seen and the bacteria had pursued their way towards the middle of the filaments. The filament tips eventually were disintegrated and bacterial infiltrates were found in between necrotic tissue. Bacteria further invaded the base of the filaments, to ultimately colonize and break down the complete gill filament. Chondrolysis was a dominating feature in the last stages of disease coincided by infiltration of massive clusters of bacterial cells. When necrosis became generalized and severe oedema was evident, the lesions became visible macroscopically as bilaterally whitish discolouration of the gill tissue.

In 75% of the carp inoculated with the LV-isolate, bacterial cells were also able to attach to the epithelium of the filament tips from the first SP onwards and subsequently moved downwards to the middle of the filaments at the third and fourth SP. In contrast, at the fifth and following SPs, the gill sections of only 25% of the fish displayed bacterial cells. Nevertheless, the bacterial titres retrieved from the gill tissue remained high for all carp inoculated with the LV-isolate up until the last SP which seems inconsistent with the histological findings. This may signify that the bacterial cells were no longer as firmly attached to the gill tissue as they were during the first four SPs. Indeed, as described before [12,16], the bacteria might be part of the aqueous biofilm covering the gill tissue and hence be noted by bacteriological examination when plating out the gill sample. However, during processing for histological examination, they might have been washed away as this technique involves several washing steps hence visualizing only firmly attached bacteria. The fifth SP seems to be the turning point for the majority of carp to head for surmounting the LV-isolate *F. columnare* infection with colonization halted at the filament middle section, and bacterial cells allegedly being less firmly attached to the gills. In contrast, the gill tissue of the few carp succumbing to the disease at that time-point (and later) exhibited bacterial colonization over the entire filament length with necrosis of the gill lamellae as a result. Lysis of the cartilaginous tissue was never encountered following exposure to the LV-isolate, which stands in shrill contrast to what was observed in the gill sections of fish challenged with the HV-isolate.

The findings for the LV-isolate challenge both in carp and rainbow trout are intriguing as the bacterial cells seem to be able to colonize the gill tissue, albeit to a significantly lesser extent than those of the HV-isolate. However, it appears that they cannot maintain a firm grip on this vital organ. The latter needs to be slightly toned down for the LV-isolate in carp where the gills of a few fish were colonized by *F. columnare* over the entire filament length. Why these few fish ultimately succumbed to the challenge with the LV-isolate and the majority of fish survived, warrants further research, as this might provide most relevant information concerning the susceptibility to this increasingly important disease.

In natural outbreaks of edible and ornamental fish, the same clinical and pathologic features have been perceived. Fish were described to firstly lose appetite, swim at the water surface with rapid opercular movements to finally lose balance and succumb to the disease. Acute to chronic massive mortality with [6,25,26] or without [25] macroscopic visible skin or gill lesions have been described. Histological examination of the affected gill tissue could reveal oedema, distortion of lamellae and clusters of bacterial cells situated at the filament tips [26] and bases [25]. In some cases, a total loss of gill architecture was observed [26].

Although skin lesions have been described in natural outbreaks of columnaris disease in salmonids [6], no skin lesions were observed in this study. Most probably, the disease pattern evolved too fast for skin lesions to be discernible, as the latter usually appear in more chronic cases of columnaris disease [1]. In the present studies, we aimed to mimic the natural situation as much as possible. Therefore, carp were inoculated with *F. columnare* strains isolated from carp, and rainbow trout with strains from trout. Our results therefore do not allow concluding whether the temporal differences in infection observed between rainbow trout and carp are fish species related or rather *F. columnare* strain dependent.

Virulence is determined by more than the process of colonization. Upon TEM examination of the carp and rainbow trout gill sections of fish inoculated with the HV-isolates, OMV were discerned surrounding the majority of bacterial cells. These OMV were markedly less frequent in the vicinity of the bacterial cells upon inspection of gill sections of fish exposed to the LV- carp isolate. The production and composition of OMV seem to be influenced by ambient factors that microbes sense inside the host during normal cell growth [27] and not during cell lysis [28]. OMV have been described to contain biologically active proteins important for nutrient acquisition, co-aggregation of bacteria and biofilm development, tissue lysis and virulence [29-31]. These bacterial “bombs” have been detected in *F. columnare* after in vitro research [32,33], but, as far as we know, they have never before been described in vivo.

The findings in the present study strongly point towards biofilm development as has been demonstrated for *F. columnare* in in vitro experiments [34]. These in vivo findings comply with the demonstration of genes in *F. columnare* encoding biofilm formation by the research group of Tekedar et al. [35]. The recurring features stressing biofilm formation potentially being an important stage in the pathogenicity of *F. columnare*, warrant further investigation.

Another important parameter to investigate in the theme of virulence, is the myriad of ways adopted by the pathogen to escape the host’s immune defence. A remarkable and hitherto unexplained lack of inflammatory response is typical of *F. columnare* infections, especially in the early stages, allowing progression of the infection. Apoptosis constitutes a cell death programme with a notably non-inflammatory outcome, which may provide an explanation for the impaired inflammatory response and the acuteness with which columnaris disease can strike. In the present study, TUNEL and caspase-3 staining showed that significantly higher apoptotic cell counts in the gill tissue of fish challenged with the HV- or LV-isolate compared to the control animals and in some cases even between fish gills challenged with either one of the bacterial isolates were perceived. Apoptosis mostly affected epithelial cells and only occasionally goblet cells. Sun et al. demonstrated an up-regulation of apoptosis pathways in the early stages after a challenge with *F. columnare,* which corresponds to our findings [15]. The initiation of an increase in apoptotic cells following exposure to *F. columnare* – as now also demonstrated morphologically – remains enigmatic [15]. Although different techniques for apoptosis detection are available, none of these are entirely specific or all-inclusive. Therefore, it was chosen to combine different techniques in our apoptosis research. The TUNEL assay was used, because it detects DNA fragmentation. However, DNA fragmentation has also been described to occur in oncosis (cell death with swelling) [36]. Therefore, the immunohistochemical detection of caspase-3 was also applied. It is generally accepted that the latter technique is entirely specific for apoptosis, although apoptosis can be independent of caspase-3 [37].

The presence of EGC or mast cells in teleosts may be demonstrated by using H&E and Giemsa stainings [38,39]. These EGC were described to stain negative using PAS [38]. However, precise and fully documented data on the staining characteristics of EGC remain scarce, while information on the impact of the organ and/or fish species and the effect of histological processing is almost fully lacking. In the current experiment, the granules of the EGC clearly stained PAS-positive in the gill tissue of carp. These cells were negative on alcian blue and toluidine blue stained sections (data not shown), with the well-delineated granules and staining features clearly differing from what was observed for the goblet cells. EGC have been associated with host defence actions towards bacteria [39] and parasites [39,40], although their exact role and the events they elicit are far from fully known. A significantly higher amount of these cells was noted in the carp gill tissue following exposure to the HV- as compared to the LV-isolate and control groups. EGC may be mobilized towards regions attacked by insults with acute tissue damage causing mast cell/eosinophilic granular cell degranulation and release of mediators of inflammation [39]. Degranulation of EGC was also noted in the carp in sites where tissue damage was visible. Whether this was favourable or disadvantageous for the host at this stage, is unclear. As this is the first time that a marked mobilization of EGC at the level of the gill tissue following *F. columnare* challenge is described, this phenomenon justifies further research into the function and significance of these cells in the pathogenesis of columnaris disease.

In this study, both fish species showed an increase in the total amount of producing goblet cells following challenge with either one of the *F. columnare* isolates compared to the control group, but only the carp showed significantly more producing goblet cells. A similar increase in goblet cells was seen after exposure to various parasites and bacteria [41,42].

Interestingly, gill mucus cell histochemistry demonstrated an increase of neutral mucin production in carp and a significant increase of PAS-positive cell numbers following challenge with the HV-isolate as compared to the control animals. A similar mucus shift has been described in Atlantic salmon after challenge with the causative agent of amoebic gill disease [41]. After exposure to the HV- or LV-isolate in carp, a significant increase in AB-positive productive goblet cells was also observed compared to the control animals. An increased number of goblet cells containing acid glycoconjugates has been noted in skin mucus of carp after exposure to water containing a high load of non-pathogenic bacteria [42]. Although not significant, an upward trend in acid mucus production was also noted in rainbow trout following challenge with the HV-isolate. Rainbow trout in general have been described to produce less acid mucins compared to other fish species such as Atlantic salmon [40], which might explain why the observed differences were not statistically significant.

The question arises how the mucus changes observed in this study are elicited and how these may impact both the pathogen and the host. A distinct possibility is that the bacterium itself can alter the composition of the mucins. This has been described for *Helicobacter pylori* infections in the acid environment of the stomach where *H. pylori* uses urea hydrolysis to elevate the pH of its environment. The elevation of pH to neutral transforms the viscoelastic mucin gel to a viscous liquid [43], enabling the helical cell-shaped bacterium to swim faster in the viscous solution [44]. It is unknown whether *F. columnare* can steer the pH of mucus as, to our knowledge, this is unknown for any gill-disease eliciting organism. A consistent finding throughout this study is the fact that *F. columnare* bacteria were immediately surrounded by a PAS-positive matrix whilst being encased by AB-positive mucins. As an acid environment has been described to be adverse for *F. columnare* bacteria [45], it is most tempting to speculate that the more neutral mucins in the immediate vicinity of the bacterial cells presumably are self-produced whilst the acid AB-positive mucus would be secreted by the host as a self-defence mechanism. Indeed, acid AB-positive mucins at pH 2.5 contain negative charges which reduce bacterial binding and are missing in PAS-positive mucins. In view of the importance of mucus in the fish’s arsenal to combat disease, again, this finding should be further investigated.

In conclusion, the present study has revealed that, following immersion challenge, adhesion and aggregation of HV- *F. columnare* isolates adhere first at the tips of the gill filaments before the bacterial cells pursue their way to the middle and base of the filament, and eventually colonize the complete filament. Rainbow trout showed more focal tissue destruction compared to complete gill filament disintegration being most conspicuous in carp. Moreover, lysis of the cartilaginous tissue was perceived in carp. The production of OMV merits further attention, as these structures have been described to play a role in virulence and tissue lysis and have, as far as we know, never before been described in *F. columnare* in vivo. Furthermore, biofilm formation was discerned with bacterial cells wrapped in a PAS-positive matrix while being encased by AB-positive mucus. The observed shifts in goblet cells both quantitatively and qualitatively point towards the complex yet intriguing interplay of *F. columnare* with the gill mucus again warranting further research. The perceived increase in apoptotic cells and the higher EGC counts conclude the list of conspicuous features that provide pointers for future research on the pathogenesis of columnaris disease.

## Additional files

Additional file 1:
**Gill section of a control carp at SP 7.** The normal gill tissue structure is shown with parallel gill filaments (F) and sprouting from the filaments, intact gill lamellae (L) displayed (H&E, bar = 50 μm). Additional file 2:
**Gill section of a control carp at SP 7.** The normal gill tissue structure is shown with parallel gill filaments (F) and sprouting from the filaments, intact gill lamellae (L) displayed (PAS/AB, bar = 50 μm). Additional file 3:
**Gill tissue of a control carp.** One gill filament (upper structure) and the parallel orientated gill lamellae sprouting from this gill filament are visible (SEM, bar = 10 μm). Additional file 4:
**Gill tissue of a control trout.** The tops and middle parts of gill filaments are shown and sprouting from the latter, parallel ranked gill lamellae can be distinguished (SEM, bar = 100 μm). Additional file 5:
**Carp gill after inoculation with HV-isolate at SP 3.** Detail of densely packed bacterial cells wrapped between mucus and necrotic debris (SEM, bar = 2 μm). Additional file 6:
**Carp gill after inoculation with HV-isolate at SP 7.** A detail of densely packed bacterial cells wrapped between mucus and necrotic debris is shown, causing the gill lamellae to be covered (SEM, bar = 5 μm).
