# Supplementary figures and images for: Interactions of highly and low virulent Flavobacterium columnare isolates with gill tissue in carp and rainbow trout
Source: Vet Res. 2015 Mar 6;46:25. doi: 10.1186/s13567-015-0164-5 (PMC4350652; doi:10.1186/s13567-015-0164-5)

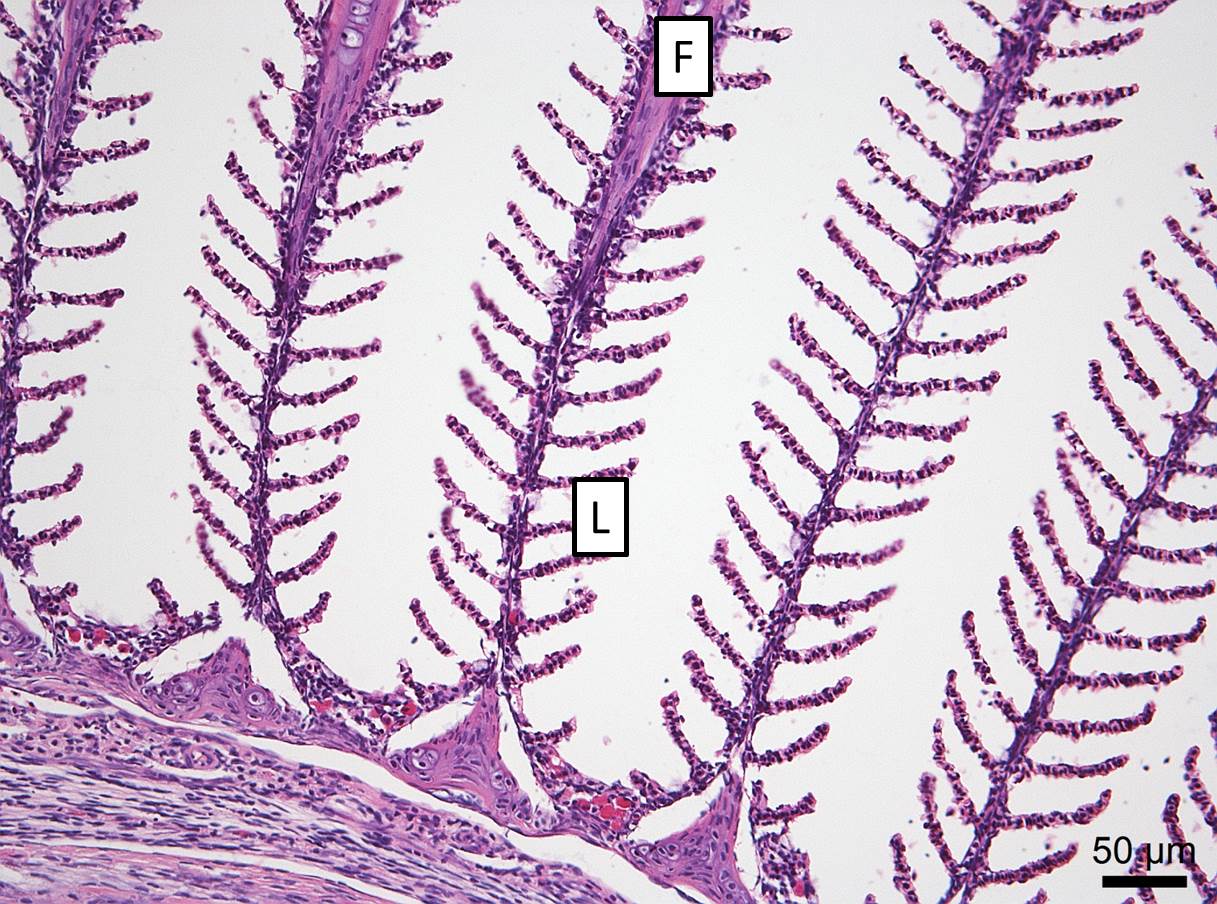

Supplement: Additional file 1: — Gill section of a control carp at SP 7. The normal gill tissue structure is shown with parallel gill filaments (F) and sprouting from the filaments, intact gill lamellae (L) displayed (H&E, bar = 50 μm). [file 13567_2015_164_MOESM1_ESM.jpeg]

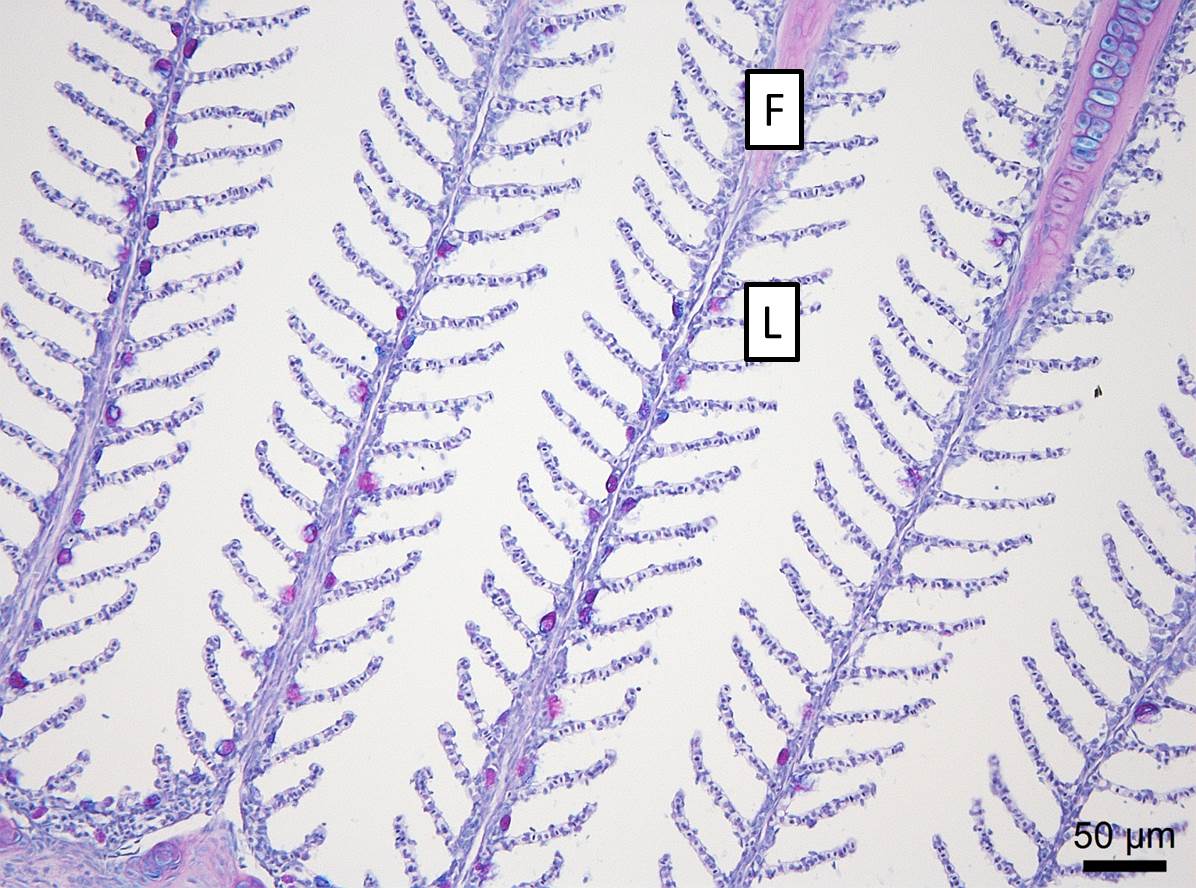

Supplement: Additional file 2: — Gill section of a control carp at SP 7. The normal gill tissue structure is shown with parallel gill filaments (F) and sprouting from the filaments, intact gill lamellae (L) displayed (PAS/AB, bar = 50 μm). [file 13567_2015_164_MOESM2_ESM.jpeg]

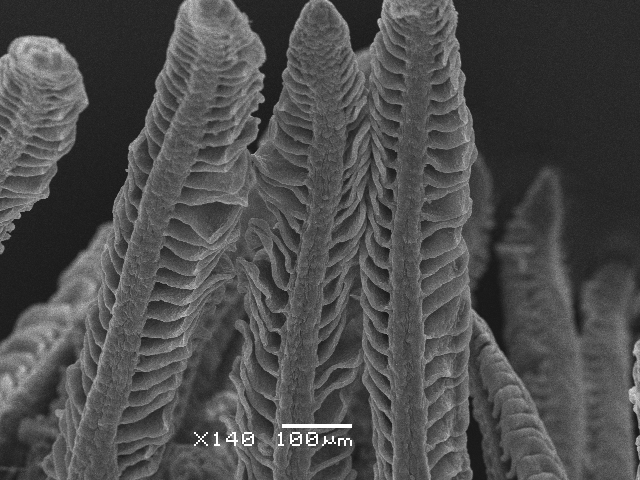

Supplement: Additional file 4: — Gill tissue of a control trout. The tops and middle parts of gill filaments are shown and sprouting from the latter, parallel ranked gill lamellae can be distinguished (SEM, bar = 100 μm). [file 13567_2015_164_MOESM4_ESM.tiff]
